# Signet Ring Cell Histology Is Not an Independent Predictor of Poor Prognosis After Curative Resection for Gastric Cancer: A Propensity Analysis by the KLASS Group

**DOI:** 10.1097/MD.0000000000000136

**Published:** 2014-12-12

**Authors:** Jung Ho Shim, Kyo Young Song, Hyung-Ho Kim, Sang-Uk Han, Min-Chan Kim, Woo Jin Hyung, Wook Kim, Hyuk-Joon Lee, Seung Wan Ryu, Gyu Seok Cho, Seong Yeob Ryu

**Affiliations:** From the Department of Surgery (JHS, KYS, Seoul St. Mary's Hospital, College of Medicine, The Catholic University of Korea, Seoul; Department of Surgery (H-HK), Seoul National University Bundang Hospital, Seoul National University College of Medicine, Seongnam, Korea; Department of Surgery (S-UH), Ajou University School of Medicine, Suwon; Department of Surgery (M-CK), Dong-A University College of Medicine, Busan; Department of Surgery (WJH), Institute of Gastroenterology, Yonsei University College of Medicine; Department of Surgery (WK), Yeouido St. Mary's Hospital, College of Medicine, The Catholic University of Korea; Department of Surgery (H-JL), Cancer Research Institute, Seoul National University College of Medicine, Seoul; Department of Surgery (SWR), Keimyung University School of Medicine, Daegu; Department of Surgery (GSC), Soonchunhyang University Bucheon Hospital, Soonchunhyang University College of Medicine, Bucheon; and Department of Surgery (SYR), Chonnam National University Medical School, Gwangju, Korea.

## Abstract

Whether signet ring cell (SRC) histology carries a worse prognosis than other forms of gastric adenocarcinoma has been questioned. The present study investigated the differences in clinicopathologic features and survival between SRC and non-SRC adenocarcinoma. The prospectively collected data of 2643 patients who had undergone curative gastrectomy between 1998 and 2005 by 10 surgeons were reviewed. Additionally, we employed analysis of covariance, propensity-score risk adjustment, and propensity-based matching to account for possible selection bias. The baseline characteristics of prematched patients with SRC or non-SRC adenocarcinoma histology differed: SRC presented in younger patients and less often in men, was more likely found in the middle stomach, and was more likely to be Stage I. After applying the propensity-score strata and propensity-score matching, there was no difference in the baseline characteristics, and SRC was not an independent risk factor for mortality in the same stage. SRC is not an independent predictor of poor prognosis after curative resection for gastric cancer in Korea.

## INTRODUCTION

Signet ring cell (SRC) histology is defined based only on microscopic characteristics described by the World Health Organization (WHO),^1^ and not on its biological behavior. The WHO classifies SRC as an adenocarcinoma, the predominant component of which (>50% of the tumor) consists of isolated or small groups of malignant cells containing intracytoplasmic mucins. SRC has been variously designated as an “undifferentiated type” by the Japanese Research Society of Gastric Cancer,^2^ a “diffuse type” by Lauren,^3^ an “infiltrative type” by Ming,^4^ and “high grade” by the WHO and the International Union Against Cancer.^5^

There have been many studies on the clinicopathologic characteristics of SRC carcinoma and its prognostic significance, although the results are controversial. In some of those investigations, SRC was associated with better prognosis,^6^ whereas others^7^ found no difference in 5-year survival between patients with SRC and those with other types of gastric cancer. Moreover, some reports^8^ revealed even a worse prognosis for SRC than for the other types. Those controversies can be partially explained by differences in tumor biology between countries or centers. In any case, the results of most comparative studies conducted to date are of only limited significance, because of selection biases as well as confounding factors arising from their retrospective nature. Notably, there have been no comparative analyses of SRC versus non-SRC adenocarcinoma based on a large multicenter database in Korea.

Therefore, to investigate the differences in clinicopathologic characteristics and treatment outcomes between SRC and non-SRC adenocarcinoma, we utilized a large multicenter database from the Korean Laparoscopic Gastrointestinal Surgery Study (KLASS) group and performed a propensity analysis to confirm the validity of this observational study.

## PATIENTS AND METHODS

### KLASS Group

To provide background data for a multicenter randomized clinical trial comparing open with laparoscopy-assisted gastrectomy^9^ (KLASS trial, NCT00452751), a retrospective multicenter study was carried out in Korea that involved 3053 patients who had undergone gastrectomy by 10 surgeons from 10 institutions between April 1998 and December 2005.^10^ All of the participating surgeons were personally responsible for obtaining written informed consent from their patients. The Institutional Review Board of each participating institution approved this study.

All of the data were collected in the same database format after reviewing medical records from each institution. The data included patient clinicopathologic demographics, surgical–procedural details, surgical and postoperative outcomes including complications, as well as long-term survival outcomes. Tumor depth, nodal classification, and stage were classified according to the American Joint Committee on Cancer Staging Manual (7th edition).^5^

### Patient Sample

From a group of 3053 patients with SRC and non-SRC adenocarcinomas, we excluded 410 individuals on the basis of the following criteria: noncurative resection (n = 27), carcinoid histology (n = 2), dysplasia histology (n = 2), completion gastrectomy (n = 5), and unknown histology (n = 374). This left us with a final analytic sample of 2643 patients.

### Statistical Analysis

Categorical variables were expressed as the n (%) and continuous variables as the mean ± standard deviation when the data followed a normal distribution, or as the median [interquartile range (IQR)] when the distribution departed from the norm. The categorical variables were compared using either the χ^2^ test or the Fisher exact probability test, the means by the Student *t* test, and the medians by the Wilcoxon rank sum test. Overall survival curves were drawn using the Kaplan–Meier method, and the log-rank test was utilized to evaluate the statistical significances of the differences. The prognostic significances of the demographic and pathologic characteristics were determined by univariable and multivariable Cox proportional hazard regression analyses.

We estimated the propensity using multivariable logistic regression to model a dichotomous SRC or non-SRC outcome for the 1548 patients in the sample (owing to missing covariate data, 1095 of the 2643 patients could not be assigned a score). On the basis of these propensity scores, we performed a rigorous adjustment for differences in the patients’ baseline characteristics. A full nonparsimonious model that included 14 covariates was developed, which yielded a c statistic of 0.8, indicating a good ability to differentiate between SRC and non-SRC. The success of the propensity-score modeling was determined by whether the baseline characteristics of the SRC and the non-SRC patients were balanced within a quintile of the propensity score. To that end, one-to-one matching was performed by the Greedy matching method using a macro available online in the public domain (http://www2.sas.com/proceedings/sugi26/p214-26.pdf), after which the balance of the SRC type was evaluated using the Wilcoxon rank sum test for continuous variables and the χ^2^ or Fisher exact test for categorical variables. A *P* value of <0.05 was considered statistically significant. Following the propensity-score estimation, we performed 3 techniques of Cox proportional hazard regression: stratification (quintiles), inverse probability of treatment weighting (IPTW), and SRC/non-SRC matching. We considered a 2-sided *P* < 0.05 value to be statistically significant. All statistical analyses were performed using SAS software (SAS Institute, Inc, Cary, NC).

## RESULTS

### Clinicopathologic Characteristics

The patient demographics are listed in Table 1. Of the 2643 participants in this study, 377 (14.3%) had SRC carcinoma and 2266 (85.7%) had non-SRC adenocarcinoma. SRC was found more commonly in younger patients, a smaller proportion of whom were male (50.66% vs 69.42%, *P* < 0.001).

**TABLE 1:**
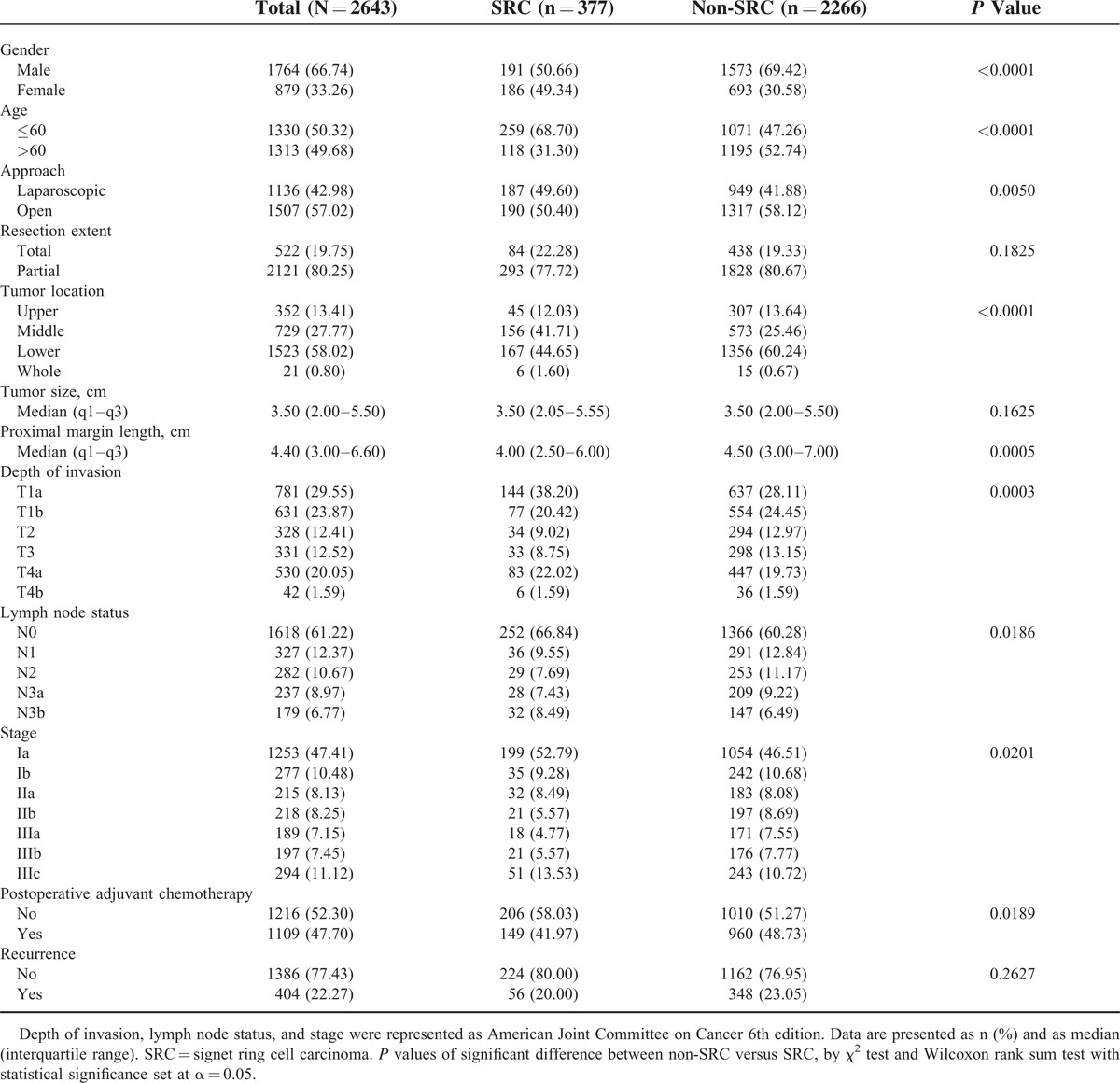
Demographics of Patients With SRC Versus Non-SRC

There was a higher incidence of SRC in the middle third of the stomach, and this was more often associated with the depressed type of lesion. The detection rate of early-stage gastric cancer (T1) was higher in SRC than in non-SRC adenocarcinoma (58.6% vs 52.6%, *P* = 0.0289).

### Survival

Over the median follow-up duration of 69.8 months (range: 0–141.9), 627 patients (23.72%) died. The overall survival rates comparing all stages of SRC with those of non-SRC adenocarcinoma were not significantly different (80.1% vs 75.6%, *P* = 0.0591) (Table 2).

**TABLE 2:**
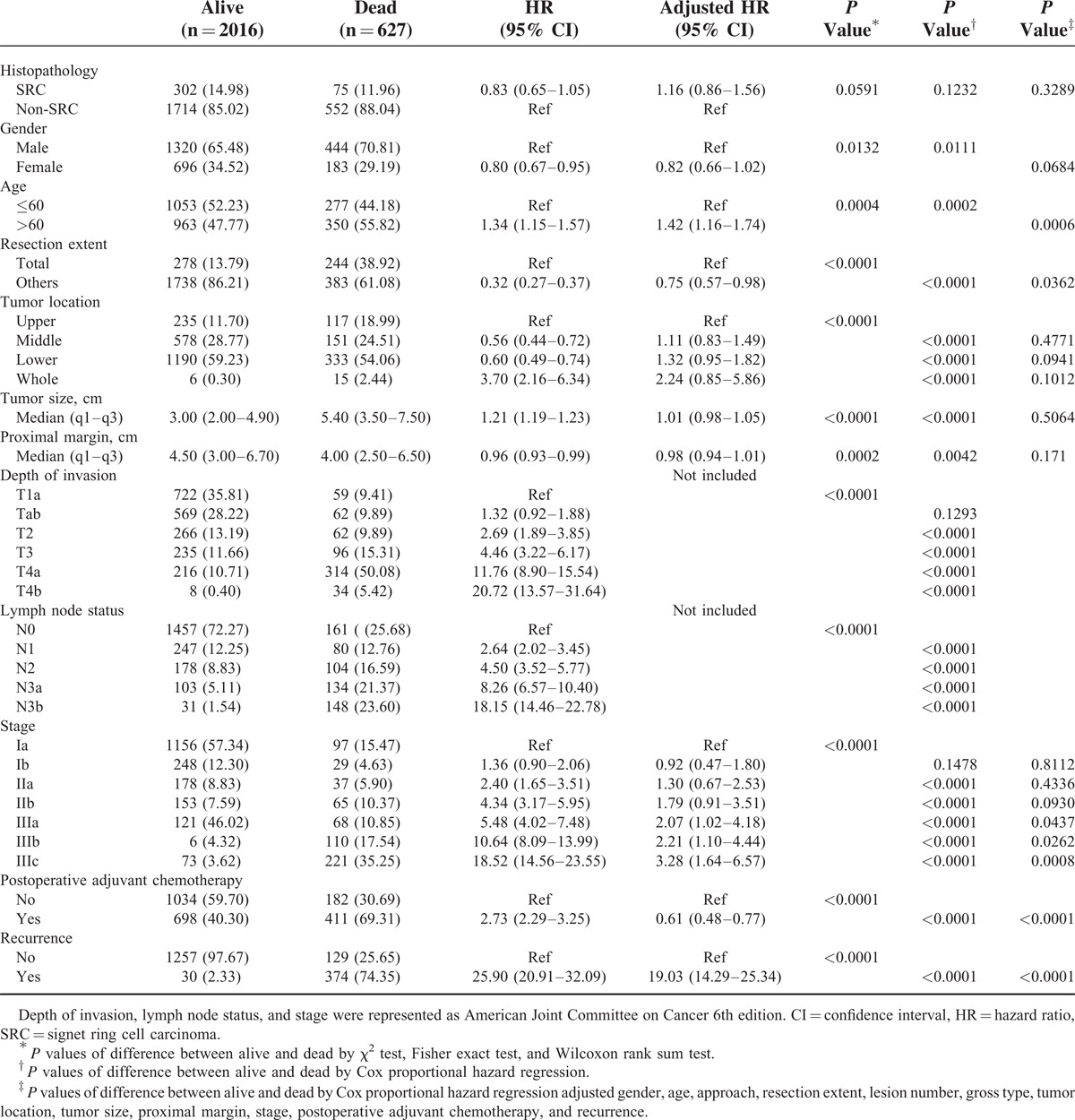
Unadjusted and Adjusted Risk Factors With Mortality

Neither were there any significant survival differences between any of the respective SRC and non-SRC adenocarcinoma stages (Figure 1 ).

**FIGURE 1:**
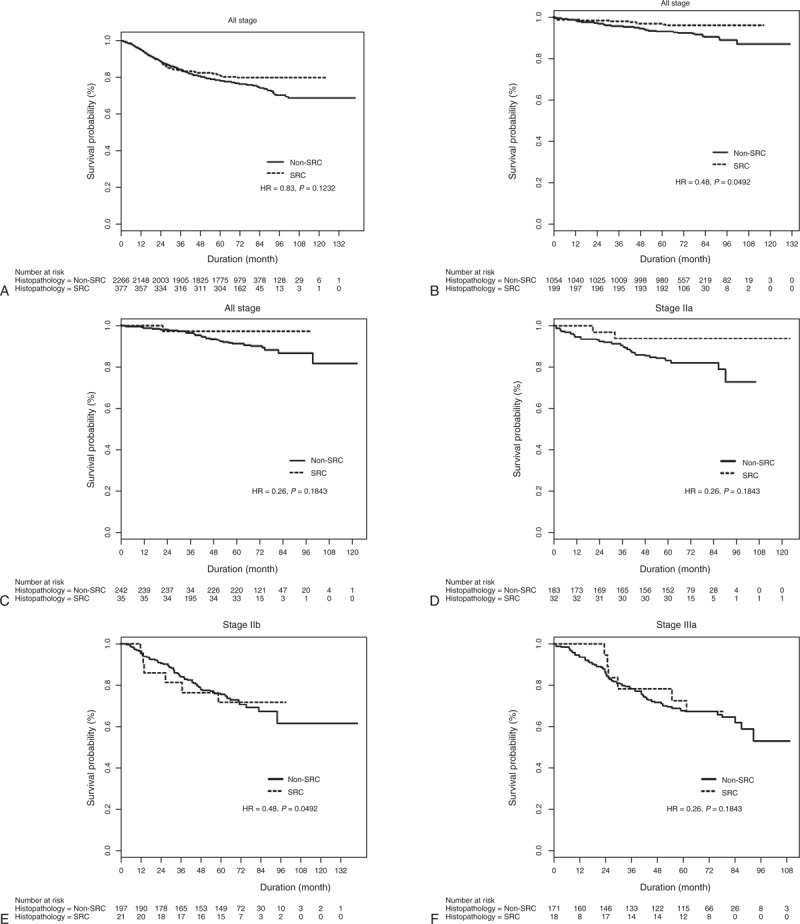
Overall survival rates of patients with SRC and non-SRC histology for (A) all stages, (B) American Joint Committee on Cancer, 7th edition Stage Ia, (C) Stage Ib, (D) Stage IIa, (E) Stage IIb, (F) Stage IIIa, (G) Stage IIIb, and (H) Stage IIIc tumors. HR = hazard ratio, SRC = signet ring cell.

**FIGURE 1 (Continued):**
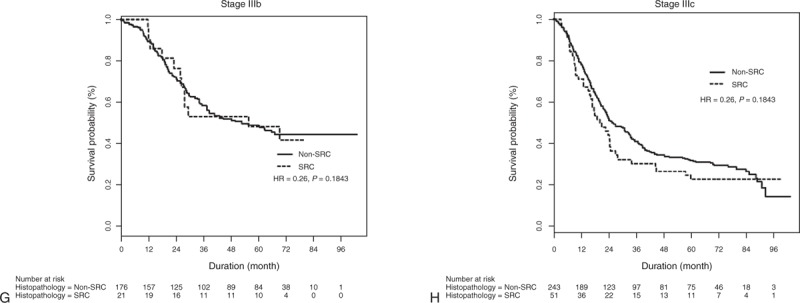
Overall survival rates of patients with SRC and non-SRC histology for (A) all stages, (B) American Joint Committee on Cancer, 7th edition Stage Ia, (C) Stage Ib, (D) Stage IIa, (E) Stage IIb, (F) Stage IIIa, (G) Stage IIIb, and (H) Stage IIIc tumors. HR = hazard ratio, SRC = signet ring cell.

### Predictors of Mortality

An unadjusted Cox proportional hazard regression analysis showed that gender, age, resection extent, gross type, tumor location, proximal margin length, lymph node status, depth, and stage was significantly associated with mortality (Table 2). However, SRC was not a risk factor for mortality (hazard ratio [HR]: 0.83; 95% confidence interval [CI]: 0.65–1.05; *P* = 0.1232). The multivariable results from the analysis also are listed in Table 2. Age >60 at diagnosis (multivariable Cox HR: 1.42; 95% CI: 1.16–1.74; *P* = 0.0006) and increasing stage were independently associated with mortality. However, SRC was not an independent predictor of mortality (multivariable Cox HR: 1.16; 95% CI: 0.89–1.56; *P* = 0.3289).

### Prognostic Impact of SRC in Propensity-Matched Patients

We successfully matched 216 patients who had SRC with 216 patients who had non-SRC adenocarcinoma on the basis of their propensity scores, which were estimated from variables pertaining to their clinicopathologic characteristics. Table 3 shows that, on the histological basis, the main characteristics of these patients did not differ between the 2 groups. Indeed, relative to the entire population, these patients were well matched. When using quintiles of the propensity scores as strata in the Cox proportional hazard model, the hazard ratio for mortality risk between SRC and non-SRC was 0.92 (95% CI: 0.68–1.25; *P* = 0.5979) (Table 4). After IPTW adjustment, death (HR: 1.14; 95% CI: 0.95–1.37; *P* = 0.1605) did not differ between the 2 groups. Among the propensity-matched 432 patients with gastric adenocarcinoma, overall survival was compared between SRC and non-SRC adenocarcinoma, and no differences were observed overall (HR: 0.83; 95% CI: 0.58–1.19; *P* = 0.3078) or for any stage (Figure 2 ).

**TABLE 3:**
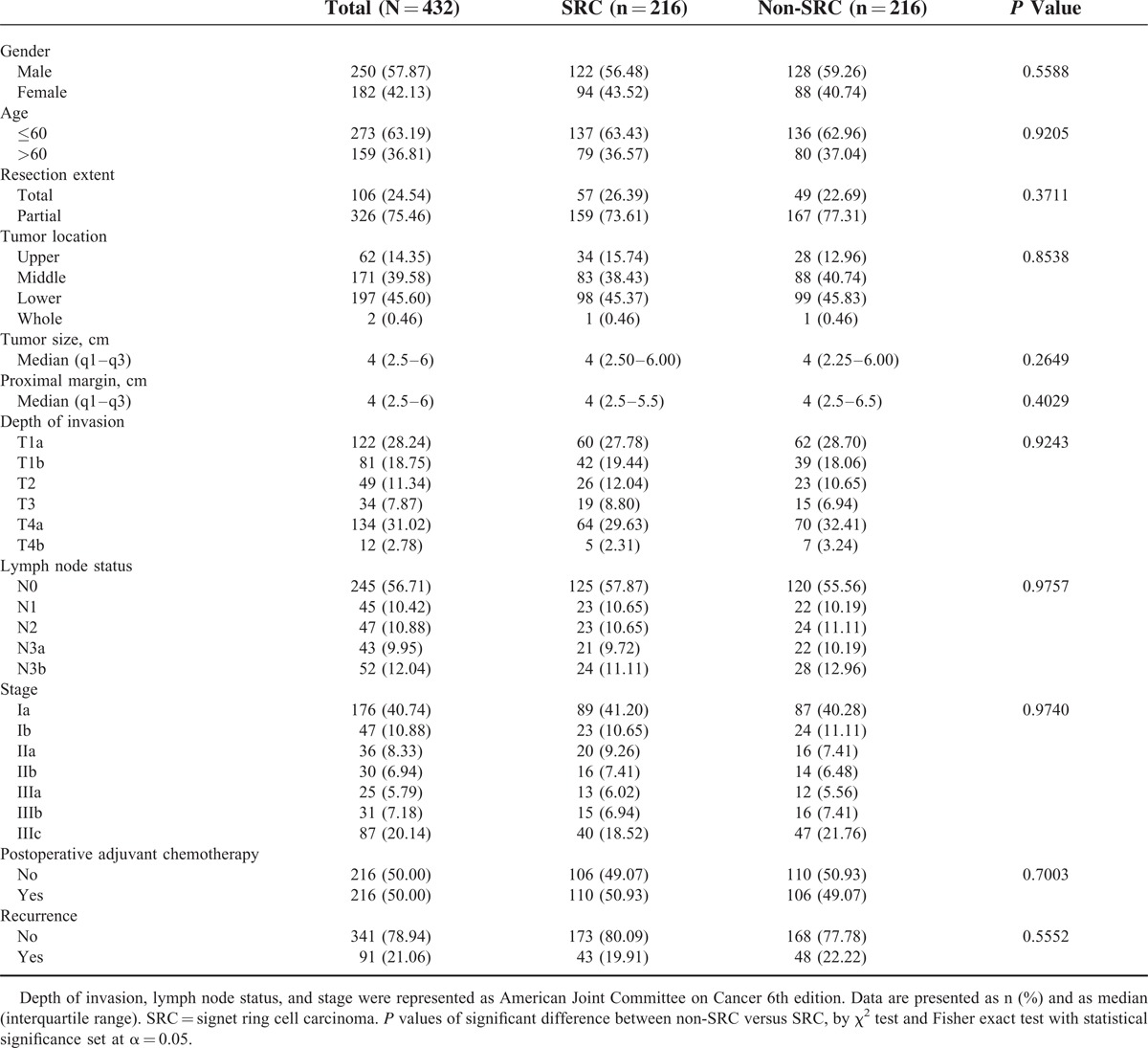
Demographics of Patients in Propensity Matching

**TABLE 4:**
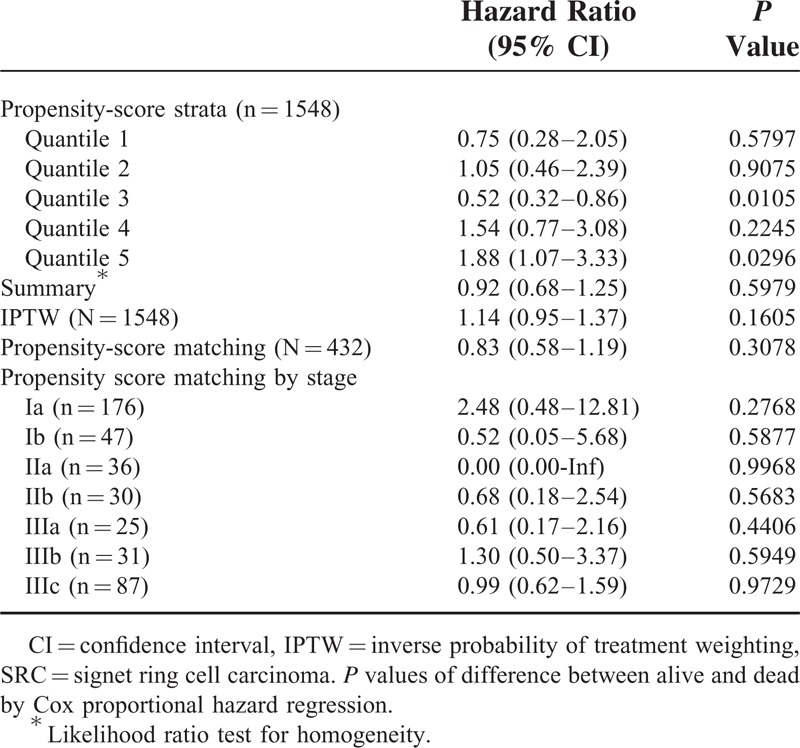
Association SRC and Mortality in Propensity-Score Analysis

**FIGURE 2:**
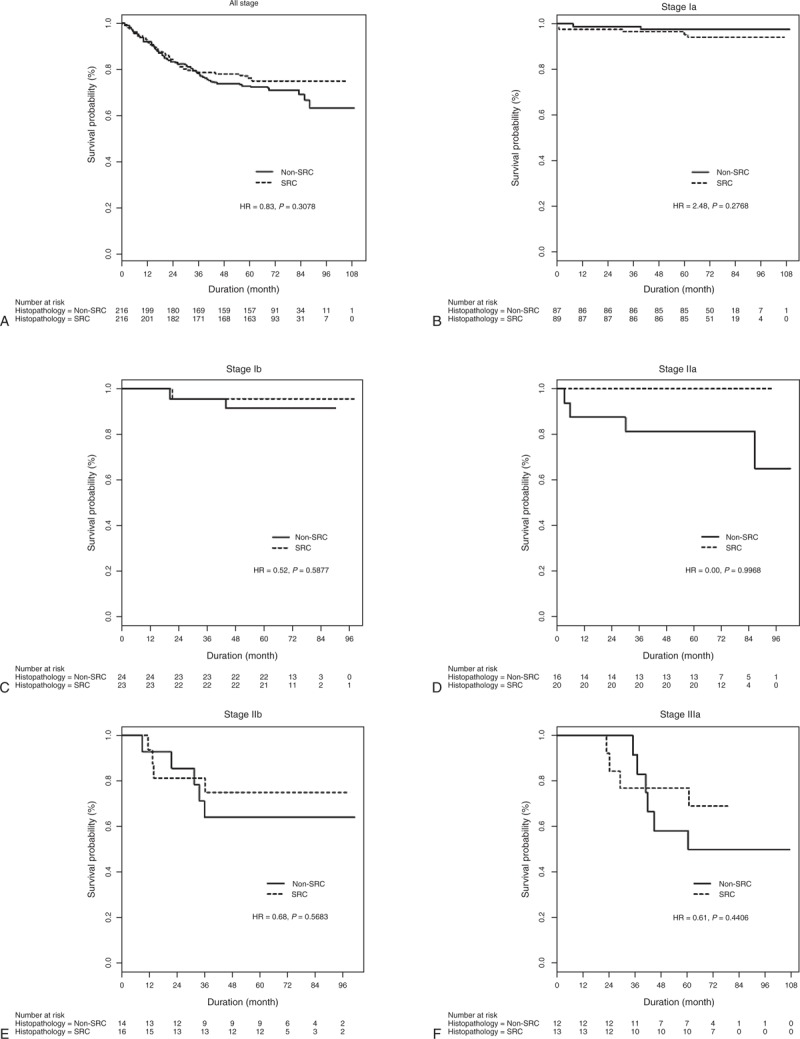
Overall survival rates of propensity-matching patients with SRC and non-SRC histology for (A) all stages, (B) American Joint Committee on Cancer, 7th edition Stage Ia, (C) Stage Ib, (D) Stage IIa, (E) Stage IIb, (F) Stage IIIa, (G) Stage IIIb, and (H) Stage IIIc tumors. HR = hazard ratio, SRC = signet ring cell.

**FIGURE 2 (Continued):**
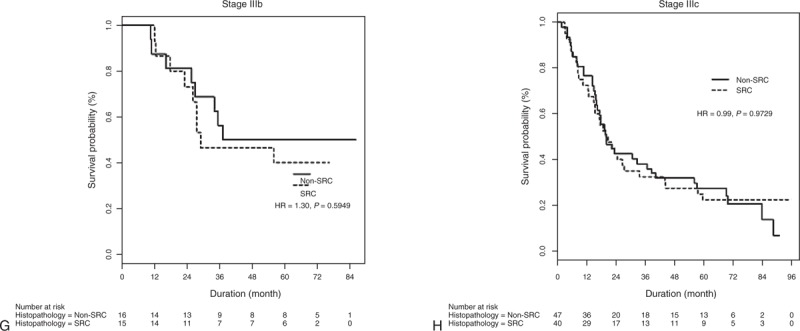
Overall survival rates of propensity-matching patients with SRC and non-SRC histology for (A) all stages, (B) American Joint Committee on Cancer, 7th edition Stage Ia, (C) Stage Ib, (D) Stage IIa, (E) Stage IIb, (F) Stage IIIa, (G) Stage IIIb, and (H) Stage IIIc tumors. HR = hazard ratio, SRC = signet ring cell.

## DISCUSSION

SRC appears to occur frequently in females and more often in the middle third of the stomach. These clinical characteristics are similar to those identified in many previous studies. This notwithstanding, the reports on patient prognosis for this unique histology have been various. Specifically, comparative studies have found that SRC is associated with worse,^8^ equivalent,^11^ or better^12^ survival than non-SRC adenocarcinoma. Piessen et al^13^ founded worse survival of the SRC on the following bases: higher prevalence of peritoneal carcinomatosis and lymph node invasion on initial diagnosis, a lower R0 resection rate due to its infiltrating character, leading to more positive vertical margins despite more extensive surgery, and earlier relapse, primarily in the form of peritoneal carcinomatosis. A recent US study^14^ also found that SRC presents at later stages: a greater proportion of patients presented at Stage 4, with a more advanced tumor node metastasis stage and a higher tumor grade. Li et al^15^ made similar observations for advanced gastric cancer: advanced gastric SRC showed a proportionally higher lymph node metastasis rate and was positively associated with a significantly higher peritoneal metastasis rate. Cimerman et al^16^ characterized advanced SRC in terms of a macroscopically diffuse scirrhous appearance proceeding to peritoneal metastasis; also, it manifested, relative to non-SRC adenocarcinoma, proportionally more IIIb and IV stages.

Asian studies have reported improved or similar survival with early stages of SRC compared with non-SRC adenocarcinoma. Hyung et al,^6^ studying early gastric carcinoma, reported a significantly higher cumulative survival rate among patients with SRC histology than for those with non-SRC: the 5-year survival rates were 94.2% for SRC and 91.6% for non-SRC. Meanwhile, Kim et al^17^ found that the prognosis for SRC-type early gastric cancer (EGC) was similar to that of other histological types.

Whether SRC histology has poorer outcomes or different biological characteristics remains uncertain. There are several possible explanations for this controversy. First, most of the previous studies were of a retrospective nature and involved small patient samples at a single institute. Second, in most of those studies, SRC and non-SRC adenocarcinoma were not comparable, because of different clinicopathologic characteristics. Third, although SRC is thought to entail different biological characteristics with distinct mechanisms of carcinogenesis, the clinical impact of this histology is masked by the dominant effect of distant metastasis on prognosis. And, significantly in this regard, most of the relevant studies have included cases with distant metastasis. Especially in Western countries, SRC is frequently discovered at an advanced stage, and consequently, it typically comes with a poor prognosis. For example, it is well known that SRC carries a higher chance of early peritoneal seeding, whose feature might be related to poor survival rates. Meanwhile, in Asian populations including some representing from Korea and Japan, early gastric cancer has accounted for more than 50% of cases. That is, early-stage SRC is more readily detected in Asian than in Western patients. Therefore, in reviewing data on an Asian population, it is more beneficial to look at SRC characteristics without carcinomatosis or Stage IV disease.

We reviewed data for >3000 patients who had undergone gastrectomy from 10 leading institutions. This large-numbered cohort can be considered to be representative of the characteristic features of Korean gastric cancer, who are at a higher rate of early gastric cancer, extensive lymph node dissection, and better survival. All patients with noncurative resection had been excluded so as to remove the impact of distant metastasis on survival.

From an analytical standpoint, our findings are subject to selection bias as well as confounding with respect to mortality risk. To minimize these biases, we used propensity-score matching to investigate the differences in outcomes between SRC and non-SRC. Practically, the propensity score was estimated by logistic regression, with the treatment variable as the outcome and the background characteristics as the predictor variables. Within the propensity-score strata, the covariates were similarly distributed in the 2 groups. With propensity matching, the main characteristics of these patients did not histologically differ between the groups. According to the Cox proportional hazard regression techniques, the mortality-risk hazard ratio was not significantly different between SRC and non-SRC overall (HR, 0.92; 95% CI, 0.64–1.34; *P* = 0.6665) or for any stage.

In this study, utilizing propensity-score-matching analysis, we determined that SRC is not an independent risk factor for gastric cancer mortality in Korea. However, our results could not clarify the underlying biological difference between SRC and non-SRC adenocarcinoma. Also, the prominent high proportion of early-stage gastric cancer in our population could be the potential bias when we compare with the Western patients. In an upcoming study, therefore, the biomolecular characteristics of SRC should be explored in order to isolate its specific differences from non-SRC, such as *Helicobacter pylori* infection status, chromosomal changes, and human epidermal growth factor receptor 2 expression status.
